# Proteomics-based confirmation of protein expression and correction of annotation errors in the *Brucella abortus *genome

**DOI:** 10.1186/1471-2164-11-300

**Published:** 2010-05-12

**Authors:** Julie Lamontagne, Maxime Béland, Anik Forest, Alexandra Côté-Martin, Najib Nassif, Fadi Tomaki, Ignacio Moriyón, Edgardo Moreno, Eustache Paramithiotis

**Affiliations:** 1 Caprion Proteomics Inc., 7150 Alexander-Fleming, Montreal, Quebec, Canada; 2 Depto. Microbiología - Edificio de Investigación, Universidad de Navarra, Pamplona, España; 3 Programa de Investigación en Enfermedades Tropicales, Escuela de Medicina Veterinaria, Universidad Nacional, Heredia, Costa Rica

## Abstract

**Background:**

Brucellosis is a major bacterial zoonosis affecting domestic livestock and wild mammals, as well as humans around the globe. While conducting proteomics studies to better understand *Brucella abortus *virulence, we consolidated the proteomic data collected and compared it to publically available genomic data.

**Results:**

The proteomic data was compiled from several independent comparative studies of *Brucella abortus *that used either outer membrane blebs, cytosols, or whole bacteria grown in media, as well as intracellular bacteria recovered at different times following macrophage infection. We identified a total of 621 bacterial proteins that were differentially expressed in a condition-specific manner. For 305 of these proteins we provide the first experimental evidence of their expression. Using a custom-built protein sequence database, we uncovered 7 annotation errors. We provide experimental evidence of expression of 5 genes that were originally annotated as non-expressed pseudogenes, as well as start site annotation errors for 2 other genes.

**Conclusions:**

An essential element for ensuring correct functional studies is the correspondence between reported genome sequences and subsequent proteomics studies. In this study, we have used proteomics evidence to confirm expression of multiple proteins previously considered to be putative, as well as correct annotation errors in the genome of *Brucella abortus *strain 2308.

## Background

*Brucella *species bacteria are gram negative alpha proteobacteria superbly adapted for survival in intracellular environments. They infect a wide range of mammals, including essentially all economically important domestic mammals, many wild species, and humans. Brucellosis is the largest bacterial zoonosis in the world [1-3]. In humans, untreated brucellosis is a long lasting disease characterized by recurrent fever episodes and clinical manifestations that include spondylitis, severe headaches, joint or abdominal pain, endocarditis, and meningoencephalitis. In severe non-treated cases brucellosis can cause death [1-3].

Seven terrestrial *Brucella *species have been defined: *Brucella melitensis*, *Brucella abortus*, *Brucella suis*, *Brucella ovis*, *Brucella canis*, *Brucella neotomae *and *Brucella microti *which infect goats, cattle, pigs, sheep, dogs, desert wood rats and common voles, respectively [1,4]. Two *Brucella *species infecting marine mammals such as dolphins, whales, seals, sea lions and walrus have also been defined as *Brucella ceti *and *Brucella pinnipedialis *[5-7]. With the exception of *B. suis *biovar 3, the *Brucella *genome is encoded on two chromosomes, containing in total approximately 3,500 genes. Genome sequences from 32 different *Brucella *strains, representing all species, have been published either as complete genomes (10 strains) or as draft assemblies in NCBI (22 strains) [8-14]. The raw genome sequencing data of 78 other strains is also available in the Sequence Read Archive of NCBI. The genome sequences were very highly homologous, although regions of unique genetic material were also observed. It is possible that these regions are involved in establishing the distinct host preferences and biological behavior of the different *Brucella *species sequenced to date [15].

Unlike other pathogenic bacteria, *Brucella *virulence does not appear to be the result of relatively few virulence genes that can be transferred horizontally via plasmids, phages, or assembled in pathogenicity islands. *Brucella *also lack typical virulence factors such as exotoxins, flagella, capsules, and type III secretion systems. Rather, the pathogen's virulence appears to be an integrated aspect of its physiology. Therefore, to better understand *Brucella *virulence, we will need to better understand the *Brucella *proteome, including how it changes during the different stages of the intracellular and extracellular *Brucella *lifecycles, and how it interacts with host proteins and processes. Indeed, we have previously demonstrated that *Brucella *bacteria are capable of extensive, reversible, remodeling of their cell envelopes [16]. Furthermore, during the establishment of an intracellular infection, *Brucella *bacteria also appear able to carry out extensive, and reversible, modifications to their biosynthetic pathways and respiration in order to adapt to the changing microenvironments encountered in infected host cells [17]. This suggests that the *Brucella *proteome is considerably more dynamic than previously suspected, and that in depth proteomic analysis of the pathogen, as well as integration of these data with the available genomic information, will result in novel mechanistic and possibly therapeutic insights.

In this work we have generated a synthesis of the proteomic datasets we produced from multiple independent comparisons of *Brucella *strains either grown in media or retrieved from infected host cells. Some of this data is currently publicly available [[16,17];http://proteomicsresource.org/Default.aspx] with the remainder becoming available as part of this work. These studies were originally designed to identify experimental condition-specific differences in the *Brucella *proteome. We compiled the experimental evidence for any *Brucella *protein detected and compared the proteomic data to the available genomic data. We provide the first direct experimental evidence for the expression of 305 *Brucella *proteins, but also identified experimental evidence for the expression of five genes previously annotated as pseudogenes, and of start site errors in two other genes.

## Results and Discussion

### First experimental evidence of the expression of 305 proteins in *B. abortus *2308

Samples used for the proteomic analysis came from *B. abortus *either grown extracellularly in media or isolated from infected RAW264.7 macrophages. The extracellular samples included whole bacteria grown directly in tryptic soy broth, outer membrane preparations (blebs) [16] and cytosols. Intracellular samples consisted of viable *B. abortus *isolated at different time points post-infection from RAW264.7 macrophages [17] and of phagosomes isolated from infected murine phagocytic cells. We obtained 1704 peptides representing 621 different proteins, corresponding to approximately 20% of the predicted proteome. For 305 proteins, we are reporting the first experimental evidence of their expression in *B. abortus *2308 (Table 1). We also report genome annotation errors for two proteins, expression of ORFs annotated pseudogenes for four proteins and one correction to the sequence of another previously annotated pseudogene which allows for its full length expression. Peptide sequences corresponding to these 312 proteins are listed in Additional File 1. The peptide coverage for the 305 newly demonstrated proteins varied from 1 to 20, with an average of three peptides per protein. In order to confirm the expression of proteins identified by a single peptide, we manually validated all MSMS spectra that had a sequence assignment score smaller than 45. Forty-four of the 305 proteins were described previously as hypothetical with no putative function. When subcellular localizations were predicted using three publicly available tools [18-20], 226 proteins were predicted to be cytosolic, ten were inner membrane proteins, 25 were periplasmic, three were outer membrane proteins and the localization of 48 proteins could not be predicted (Table 1). Experimental evidence for the expression of the other 309 of the 620 proteins has been demonstrated previously by our group [16,17] and others [21-31]. It is important to note that we are reporting an analysis of the combined results of several independent experiments using the same bacterial strain and technology to acquire the data. However, each experiment was a separate comparative study designed to identify differentially expressed bacterial proteins under specific conditions per experiment. Proteins that were not sufficiently differentially expressed under the experimental conditions used would have not been identified. Thus, while our results can be used to confirm that the proteins reported were expressed, they may underestimate under what conditions they can become expressed.

**Table 1:** *B. abortus *2308 proteins for which the expression was demonstrated for the first time

Cytoplasm							
BAB1_0002	DnaN	BAB1_0855	GRX family	BAB1_1449	UDP-N-	BAB1_2149	PepS
BAB1_0022	Unknown	BAB1_0856	BolA-related		acetylmuramate	BAB1_2168	RpsO; S15
BAB1_0023	AroA	BAB1_0857	FGAM synthase II		L-alanine ligase	BAB1_2173	FabB
BAB1_0035	KdsB	BAB1_0861	PurS	BAB1_1508	CarB	BAB2_0083	Eda2
BAB1_0063	Unknown	BAB1_0864	HpcH/HpaI	BAB1_1512	CspA	BAB2_0090	GCN5-related
BAB1_0071	ArgG	BAB1_0874	AcpP	BAB1_1523	GreA		N-acetyltransferase
BAB1_0100	Putative AsnC family	BAB1_0880	HAD-like	BAB1_1528	SseA-1	BAB2_0109	Gnd
BAB1_0107	Trs-ABC (P-loop)	BAB1_0886	NN:DBI PRT	BAB1_1538	OmpR	BAB2_0160	Unknown
BAB1_0118	Unknown	BAB1_0896	ArgS	BAB1_1547	PepQ	BAB2_0162	L-carnitine
BAB1_0122	GyrB	BAB1_0898	NagZ	BAB1_1549	PrsA		dehydratase
BAB1_0139	NifU	BAB1_0918	GatB/Yqey	BAB1_1553	YchF	BAB2_0177	YafB
BAB1_0159	S30EA	BAB1_0924	AccC	BAB1_1613	Unknown	BAB2_0186	Fumarate hydratase
BAB1_0160	PtsN-like	BAB1_0933	PCRF 2	BAB1_1645	DhaK-1	BAB2_0187	Unknown
BAB1_0191	GABAtrnsam	BAB1_0943	TyrS	BAB1_1646	DhaK-2	BAB2_0191	HAD-like,
BAB1_0204	AdhP	BAB1_0949	SufC	BAB1_1655	GabD		subfamily IIA
BAB1_0215	ThiE	BAB1_0955	DeaD	BAB1_1669	PAS domain	BAB2_0198	Pseudouridine
BAB1_0216	ThiG	BAB1_0960	Trs heavy metal	BAB1_1671	TcaR		synthase
BAB1_0242	ManR	BAB1_1014	MetG	BAB1_1687	Dut	BAB2_0216	3-hydroxybutyryl-CoA
BAB1_0285	HisD	BAB1_1030	Gor	BAB1_1695	PurA		dehydrogenase
BAB1_0317	Trs arginine/ornithine	BAB1_1037	Mandelate racemase;	BAB1_1702	Phosphoglucosamine	BAB2_0246	P47K
BAB1_0331	ArgD		muconate lactonizing		mutase	BAB2_0293	Gal
BAB1_0344	Pip	BAB1_1043	Unknown	BAB1_1719	ThiE	BAB2_0295	DgoK
BAB1_0353	Unknown	BAB1_1050	FolB	BAB1_1722	Efp	BAB2_0296	KdgA
	dehydrogenase	BAB1_1077	Ach1p	BAB1_1751	Unknown	BAB2_0335	NADH:flavin oxidore-
BAB1_0416	DUF85	BAB1_1096	NifU-like	BAB1_1761	PyK		ductase/NADH oxidase
BAB1_0429	Polyprenyl synthetase	BAB1_1098	PRA-CH	BAB1_1778	FdxA	BAB2_0337	RocF
BAB1_0446	DnaJ	BAB1_1121	DNA gyrase subunit A	BAB1_1781	Unknown	BAB2_0343	Trx-2
BAB1_0447	FabI-1	BAB1_1130	ClpA/B	BAB1_1804	MarR family	BAB2_0358	Dcp
BAB1_0482	FabD	BAB1_1132	ClpP	BAB1_1810	AtpH	BAB2_0361	TypA
BAB1_0484	AcpP	BAB1_1156	KdsA	BAB1_1813	Transaldolase	BAB2_0365	FbaA
BAB1_0489	Guanylate kinase	BAB1_1157	PyrG	BAB1_1815	LeuS	BAB2_0366	RpiB/LacA/LacB
BAB1_0510	ThrC	BAB1_1161	TpiA	BAB1_1819	ACAT	BAB2_0367	TIM 2
BAB1_0525	PpdK	BAB1_1164	TrpC	BAB1_1824	PurH	BAB2_0370	EryC
BAB1_0532	Transthyretin	BAB1_1169	GltX	BAB1_1837	CynT	BAB2_0448	Unknown
BAB1_0540	Formyl transferase,	BAB1_1170	GltA	BAB1_1840	MmsA	BAB2_0457	FolD
	N-terminal	BAB1_1174	FabZ	BAB1_1872	PrfA	BAB2_0459	Pgl
BAB1_0544	DegT/DnrJ/EryC1/StrS	BAB1_1187	Endoribonuclease	BAB1_1874	LysC	BAB2_0460	Zwf
BAB1_0561	Man-6-P isomerase		L-PSP	BAB1_1879	GrxC	BAB2_0483	ShuT
	type II	BAB1_1188	GDPD	BAB1_1887	HemC	BAB2_0513	GcvT
BAB1_0570	XylA	BAB1_1205	ElaB-domain	BAB1_1895	FtsK-gamma	BAB2_0518	PutA
BAB1_0587	Unknown	BAB1_1212	BhbA	BAB1_1918	LpdA-2	BAB2_0566	AldA
BAB1_0588	ATP/GTP-binding	BAB1_1213	Unknown; conserved	BAB1_1926	SucC	BAB2_0568	Unknown
BAB1_0641	Alanine aminopep-	BAB1_1223	AlaS	BAB1_1936	GloB	BAB2_0572	IlvE
	tidase: Neutral zinc	BAB1_1224	RecA	BAB1_1946	SecA	BAB2_0620	Unknown
	metallopeptidase,	BAB1_1233	RpsM; S13	BAB1_1970	FadB	BAB2_0642	Acyl-CoA
	zinc-binding region	BAB1_1234	Adk	BAB1_1971	EtfA		dehydrogenase
BAB1_0666	DapA	BAB1_1241	RpsH; S8	BAB1_1988	HisC	BAB2_0644	Metal-dependent
BAB1_0671	RpoZ	BAB1_1242	RpsN; S14	BAB1_1993	Ppa		hydrolase
BAB1_0688	PyrC-1	BAB1_1244	RplX; L24	BAB1_2006	RegA	BAB2_0645	GatC
BAB1_0697	CysS	BAB1_1245	RplN; L14	BAB1_2016	RpmB; L28	BAB2_0646	GatA
BAB1_0718	MoaD	BAB1_1248	RplP; L16	BAB1_2023	ClpA/clpB	BAB2_0851	GuaB
BAB1_0740	Unknown	BAB1_1249	RpsC; S3	BAB1_2059	ParB	BAB2_0961	DapA
BAB1_0775	AspS	BAB1_1256	RpsJ; S10	BAB1_2080	HslU	BAB2_0976	AldB
BAB1_0780	HemB	BAB1_1266	RplJ; L10	BAB1_2081	HslV	BAB2_0988	ArgB
BAB1_0787	GlyA	BAB1_1280	Unknown	BAB1_2087	HisE	BAB2_0990	Unknown
BAB1_0789	RibD	BAB1_1286	GloA	BAB1_2096	PTS system IIA	BAB2_0991	DapD
BAB1_0790	RibE	BAB1_1294	Aminotransferase		subunit	BAB2_0993	DapE
BAB1_0813	CysD	BAB1_1297	Unknown	BAB1_2109	AccD	BAB2_1009	MgsA
BAB1_0817	Unknown; conserved	BAB1_1376	UreA	BAB1_2133	Unknown	BAB2_1012	DapB
BAB1_0826	NuoE	BAB1_1408	IlvB	BAB1_2134	SMP-30	BAB2_1013	Gpm
BAB1_0842	ProS			BAB1_2135	Glutathione synthetase		

**Inner membrane**							

BAB1_0400	Unknown	BAB1_1283	DUF192	BAB2_0261	RecA	BAB2_0877	Binding-protein-
BAB1_0425	NhaA	BAB1_1703	FtsH	BAB2_0709	FtsK-alpha		dependent transport
BAB1_0542	WbkC	BAB1_1712	MotA; TolQ; ExbB	BAB2_0728	CydA		system inner
							membrane component

**Periplasm**							

BAB1_0010	Trs-ABC oligopeptide	BAB1_1118	PpiB-1	BAB2_0427	Trs-ABC spermidine/putrescine	BAB2_0697	Unknown; conserved
BAB1_0155	OstA-like	BAB1_1362	LacI			BAB2_0812	Trs-ABC oligopeptide
BAB1_0404	Unknown	BAB1_1413	DegP	BAB2_0451	Trs-ABC oligopeptide		AppA family
BAB1_0444	PdxH	BAB1_1890	YciI-like protein		AppA family	BAB2_0879	Trs-ABC spermidine/putrescine
BAB1_0739	ETC complex I	BAB1_1919	Unknown	BAB2_0593	Trs-ABC amino acid		
BAB1_0776	Unknown	BAB1_1981	TlpA	BAB2_0611	Trs-ABC amino acid	BAB2_0880	Unknown
BAB1_0881	Trs-ABC amino acid	BAB2_0374	Unknown	BAB2_0664	Trs-ABC peptide	BAB2_1109	XylF
BAB1_1117	PpiB-2						

**Outer membrane**							

BAB1_0659	Omp2a	BAB1_0707	OstA	BAB1_0963	TolC		

**Unknown localization**							

BAB1_0030	Unknown	BAB1_0991	Unknown	BAB1_1543	DUF526	BAB1_2123	RpmI; L35
BAB1_0170	GrpE	BAB1_1070	WrbA	BAB1_1559	FbcF	BAB1_2176	YaeC/NLPA lipoprotein
BAB1_0389	CcoP	BAB1_1113	Unknown; conserved	BAB1_1641	Unknown	BAB1_2186	RpsT; S20
BAB1_0413	AtpB	BAB1_1152	PdhA	BAB1_1647	FabG domain	BAB2_0207	Unknown
BAB1_0418	Unknown	BAB1_1230	RplQ; L17	BAB1_1693	bZIP	BAB2_0243	YedY
BAB1_0420	Unknown	BAB1_1232	RpsK; S11	BAB1_1728	RpmE; L31	BAB2_0269	RpsU; S21
BAB1_0453	Unknown	BAB1_1240	PplF; L6	BAB1_1749	Unknown	BAB2_0351	OsmC-like protein
BAB1_0479	RpsR, S18	BAB1_1260	RpsL; S12	BAB1_1768	Unknown	BAB2_0356	Unknown
BAB1_0627	Unknown	BAB1_1270	SecE	BAB1_1784	DUF336	BAB2_0677	Unknown
BAB1_0650	Unknown	BAB1_1341	Unknown	BAB1_1814	Unknown	BAB2_0726	YbgT
BAB1_0810	RpsI; S9	BAB1_1384	Cibk	BAB1_1858	RplU; L21	BAB2_0869	HlyD
BAB1_0830	NDH-1 subunit I	BAB1_1514	AspC	BAB1_1984	LysA	BAB2_1002	NqoB

Locus tags and descriptions of proteins are indicated and proteins are organized by predicted subcellular localization.

### Correction of five pseudogene annotations

**Figure 1:**
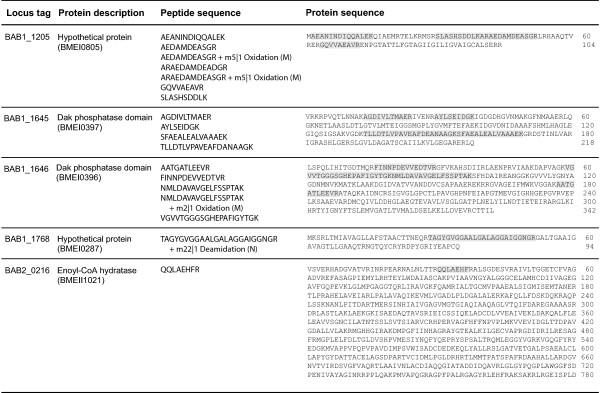
***B. abortus *2308 former pseudogenes**. Peptide sequences identified by mass spectrometry are highlighted in grey. Corresponding *B. melitensis *16 M locus tags are indicated between parentheses.

In previous studies using *B. abortus *2308, we used the genome databases available on NCBI for *B. abortus*, *B. melitensis *and *B. suis *for protein identification. More than once, we obtained peptides which matched proteins supposedly expressed only by the latter two species. Upon verification, those peptides were manually assigned to ORFs of previously annotated pseudogenes of *B. abortus *strain 2308 (NCBI taxonomy ID 359391). We therefore assembled a custom protein database which included the predicted translation sequence of all *B. abortus *2308 ORFs annotated as pseudogenes. Using this database, we were able to confirm the protein expression of five of these ORFs (Figure 1): BAB1_1205, BAB1_1645, BAB1_1646, BAB1_1768 and BAB2_0216. The MSMS spectra of the 18 peptides representing these former pseudogenes were manually validated. We thus investigated the reasons for which these genes had been annotated as pseudogenes. The genomic sequence of the cytoplasmic protein with a conserved DUF 883 domain BAB1_1205 was found to be identical to BMEI0805, its *B. melitensis *counterpart. Apart from the short length of this protein, there was no apparent reason for its pseudogene annotation (Figure 1). For BAB1_1645 and BAB1_1646 (Figure 1), the nucleotidic sequence was 99% identical to their BMEI0397 and BMEI0396 counterparts, leading to two cytoplasmic *B. abortus *2308 dihydroxyacetone kinases involved in glycerolipid metabolism that are 98% and 100% identical to the *B. melitensis *proteins, respectively. The case of BAB2_0216, which seems to be a 3-hydroxybutyryl-CoA dehydrogenase, was more complex and confusing, having a single nucleotide deletion when compared to *B. melitensis*. This deletion would lead to the silencing of the stop codon which creates two separate proteins in *B. melitensis*, BMEII1020 and BMEII1021. In *B. abortus *2308, a fusion of the two genes would generate a much larger protein. However, the start codon in the corresponding ORF of vaccine *B. abortus *S19 (BAbS19_II02060) is different from BMEII1020, and even more different from the start codon and carboxyl terminal sequence of the counterparts in *B. suis *(BSUIS_B0227), *B. ovis *(BOV_A0203), *B. canis *(BCAN_B0224) and *B. ceti *(BCETI_6000534). As a consequence, the lengths of *B. abortus *and *B. melitensis *proteins differ considerably from those of other *Brucella*. Since the BAB2_0216 peptide that we found is located in the N-terminal section of the protein (Figure 1), we are able to confirm the expression of this originally annotated pseudogene, but were unable to confirm the expression of the full length protein.

**Figure 2:**
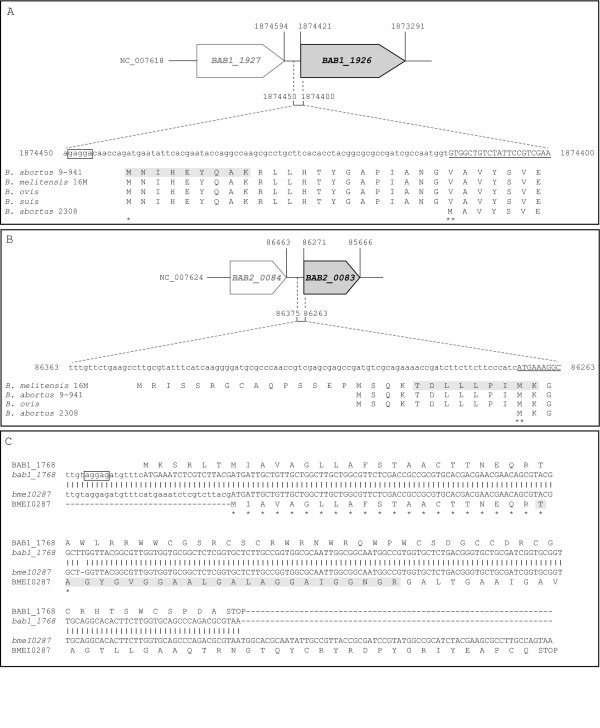
**Annotation errors in the *B. abortus *2308 genome**. (A, B) The original start codon annotation in the publicly available genome (NCBI taxonomy ID 359391) of the succinyl-CoA synthetase subunit beta (BAB1_1926, panel A) and of the KHG aldolase (BAB2_0083, panel B) are indicated by double asterisks whereas the corrected start site is indicated by a single asterisk (BAB1_1926 only). The peptides sequenced by mass spectrometry are highlighted in grey. The 5'-end of the CDS, as currently annotated, are underlined. The predicted sequence of the RBS found in proximity of the corrected start site of BAB1_1926 is boxed. Numbers next to the nucleotide sequence and the schematic gene representation indicate the position in the genome sequence (NC_007618 or NC_007624). (C) Genomic and amino acid sequences of BAB1_1768, as currently found in the publicly available genome, were aligned to their counterparts in *B. melitensis *16 M (BMEI0287). The sequence of the peptide detected by mass spectrometry is highlighted in grey. Matching nucleotides are indicated by vertical bars and matching amino acids are indicated by asterisks. The predicted sequence of the RBS found in proximity of the *B. abortus *start site is boxed.

The sequence of the BAB1_1768 pseudogene was found to be misannotated in *B. abortus *2308. The peptide sequence "TAGYGVGGAALGALAGGAIGGNGR" could not be found in the *B. abortus *2308 nucleotide-derived proteome but matched the *B. melitensis *locus tag BMEI0287. In fact, except for 1 nucleotide, the corresponding 2308 genomic sequence is identical to that of BMEI0287 (Figure 2C). In *B. abortus *2308, a single nucleotide insertion in BAB1_1768 modifies the reading frame, hence its original annotation as a pseudogene. The manually validated peptide matches *B. abortus *2308 only when the additional nucleotide is removed, indicating that the sequence for locus BAB1_1768 should be corrected (Figure 1). Also to note is the earlier start site in *B. abortus *2308, and all other species sequenced to date, when compared to *B. melitensis *16 M. We believe that the *B. abortus *2308 start site was correctly assigned in the publicly available genome given the clear presence of a ribosome binding site in position -8 of the *B. abortus *sequence.

### Correction of two start site annotations errors

Another type of annotation error identified in our studies was the erroneous assignment of gene translation start sites. For 2 proteins of *B. abortus *2308, we report the expression of manually validated peptides corresponding to the sequence found upstream of their currently annotated start sites (Figure 2). The peptide sequence "MNIHEYQAK" was first found to match the cytoplasmic *B. melitensis *succinyl-CoA synthetase subunit beta protein (BMEI0138) and then assigned manually to BAB1_1926. Sequence comparison with other *Brucella *species and strains shows that the *B. abortus *2308 protein start site is not shared with any of the subject sequences (Figure 2A). In fact, all homologues of this protein in other *Brucella *strains or species share the same start site, which is found 22 amino acids upstream of the *B. abortus *2308 site. Moreover, a ribosome binding site can clearly be mapped to position -8 of the proposed new translation start site. We therefore believe this new start site to be accurate.

The second peptide, "TDLLPIMK", was found to match the cytoplasmic *B. melitensis *keto-hydroxyglutarate-aldolase (BMEII0009) and then assigned to BAB2_0083 in *B. abortus *2308. This peptide overlaps the region upstream to the currently annotated translation start site and the first three amino acids based on the annotated translation start site (Figure 2B). Alignment of the current *B. abortus *2308 protein sequence with its counterparts in other *Brucella *strains and species indicates that the 2308 protein sequence is falsely truncated. Other start sites lead to proteins having N-terminals longer by 11, 26 or 44 amino acids. Although we cannot clearly indicate the actual start site of BAB1_1926 or BAB2_0083, we can confirm that their N-terminals are longer than currently annotated. Based on the homology of the *B. abortus *2308 genome being highest with that of other *B. abortus *strains, one can speculate that the start sites would be identical to those mapped in these strains.

### Operons

Since genes that are part of an operon are usually co-transcribed, it is possible that these genes might also be co-translated [32]. Considering all proteins identified by our studies, we were able to almost fully reconstitute one of the two ribosomal RNA operons, with all but BAB1_1237 found. Additionally, the previously mentioned BAB1_1645 and BAB1_1646 genes are predicted to be part of an operon containing 6 genes, BAB1_1645 to BAB1_1650 http://www.microbesonline.org/operons/gnc359391.html. Four of these proteins were detected in our studies, although only BAB1_1645, -46 and -48 were found in the same experimental condition.

## Conclusions

Mass spectrometry has proven to be a valuable tool to identify and correct genomic annotation errors in the study of microorganisms [33-37]. We performed a proteomics analysis of *B. abortus *2308 proteins expressed upon extracellular and intracellular growth conditions to validate existing gene predictions at the protein level, to acquire useful information on *B. abortus *2308 expressed proteins and to identify and correct inaccurately annotated ORFs. We were able to confirm the expression of over 300 previously unreported proteins and five pseudogenes, and corrected two wrongly assigned translation start sites. Taken together, these findings further demonstrate that computational genomic annotation errors can be corrected using proteomics. This will lead to improved databases and thus better protein identification and functional annotation.

## Methods

### *Brucella abortus *protein preparation for mass spectrometry analysis

Four types of *B. abortus *2308 samples were prepared: outer membranes, cytosols, intracellular bacteria isolated from infected RAW264.7 macrophages and extracellular bacteria from overnight cultures. Outer membrane samples were prepared and processed for mass spectrometry analysis as previously described [16]. Cytoplasmic fractions were prepared as described previously [38]. Briefly, bacteria grown in tryptic soy broth (Difco) in 2-liter flasks on an orbital shaker and harvested by centrifugation in sealed cups at 7,000 × g for 20 min. The thick slurry of bacteria were suspended in 10 mM phosphate-buffered saline (pH 7.2) was passed twice through a French press (Pressure Cell 40 K, Aminco; SLM Instruments Inc., Urbana, Ill.) at an internal pressure of 35,000 lb/in^2^. The homogenate was digested with 50 mg of DNase II type V and RNase A per ml (Sigma) for 18 h at 37°C and fractionated by ultracentrifugation. The cell envelopes in the bottom of the tube removed and the cytoplasmic fractions in the supernatant, filtered, lyophilized and characterized as described previously [39]. Intracellular bacteria were isolated from RAW264.7 macrophages 3, 20 and 44 hours post-infection as previously described [17]. Proteins were extracted from intracellular and extracellular bacteria using the same method and digested for mass spectrometry as previously described [17].

### Liquid Chromatography - Mass Spectrometry (LC-MS)

Peptide digests were analyzed by liquid chromatography coupled to mass spectrometry (LC-MS) as described [40]. Briefly, the samples were injected onto a reversed-phase column (Jupiter C18, Phenomenex, Torrance, CA) for HPLC separation. For LC-MS survey scans, the mass spectra were acquired over 400-1600 Da at a rate of 1 spectrum/second. Peptide sequencing was achieved by targeted and shotgun LC-MS/MS. For MS/MS scans, the mass range was 50-2000 Da, and each spectrum was acquired in 2 seconds. For LC-MS/MS, the duty cycle was one survey scan followed by one product ion scan (MS/MS).

### Protein identification

Protein identification was done by submitting LC-MS/MS spectra to Mascot software (MatrixScience, Boston, MA) and searching against custom protein databases (see below). The parameters used for the Mascot search and protein homology clustering were previously detailed [16]. No multidimensional fingerprinting method was used. Annotation for each protein was performed using ExPASy Proteomics tools http://us.expasy.org/tools/#proteome, Kegg GenomeNet Database Service http://www.genome.jp/ and literature mining of orthologous genes and proteins.

### Protein databases

The databases were composed of protein sequences obtained from the National Center for Biotechnology Information (NCBI) protein database (for *B. abortus *2308, NC_007618 and NC_007624; for *B. melitensis *16 M, NC_003317 and NC_003318; for *Mus musculus*, all protein sequences contained under taxonomy ID 10090) and of *B. abortus *2308 "pseudoproteins" corresponding to the custom translation of pseudogenes. Genomic regions corresponding to the 316 entries annotated as pseudogenes in NCBI were directly translated and added to the database. Additionally, the ORF Finder tool from NCBI was used to determine other possible protein sequences corresponding to the pseudogenes. The ORF search was done by including 0 to 200 bp upstream or downstream from these regions. All resulting ORFs spanning the entire pseudogene sequence were kept. Ribosome binding sites were mapped when possible according to the sequence described in reference [41]. A total of 471 translated protein sequences were added to the NCBI databases.

### Validation of mass spectrometry results

Sequences assigned to MS/MS spectra of peptides, which were mapped to pseudogenes or to genomic regions annotated as untranslated regions, were manually validated. For proteins identified by a single peptide, manual validation of the spectra was performed for peptide sequences having a Mascot score below 45.

### Prediction of protein localization

The localization of newly demonstrated proteins was predicted using PSORTb version 2.0.4 http://www.psort.org/psortb/index.html, CELLO version 2.5 http://cello.life.nctu.edu.tw/ and PSLpred http://www.imtech.res.in/raghava/pslpred/index.html. For a localization to be assigned, a minimum of 2 of the 3 predictions had to match.

## Authors' contributions

JL designed and coordinated the study, analyzed the data and wrote the manuscript. MB participated in the data analysis and manuscript writing. AF performed the mass spectrometry experiments and peptide validations. ACM participated in the data analysis. NN performed the protein identification steps. FT participated in the protein identification steps. IM participated in the data analysis and manuscript writing. EM participated in the data analysis and manuscript writing. EP conceived of the study and participated in manuscript writing and study coordination. All authors read and approved the final manuscript.

## Supplementary Material

Additional file 1 **Proteins newly demonstrated in *B. abortus *2308**. Each entry is represented by a gene locus tag, description of the protein and the sequences of the peptides measured. Proteins are organized by predicted subcellular localization. Click here for file

## References

[B1] Moreno E Moriyon I Dworkin M, Falkow S, Rosenberg E, Schleifer KH, Stackebrant E The Genus *Brucella* The Prokaryotes 2006 New York: Springer-Verlag 315 456 full_text

[B2] Mantur BG Amarnath SK Brucellosis in India - a review J Biosci 2008 33 539 547 10.1007/s12038-008-0072-1 19208979

[B3] Bouza E Sanchez-Carrillo C Hernangomez S Gonzalez MJ Laboratory-acquired brucellosis: a Spanish national survey J Hosp Infect 2005 61 80 83 10.1016/j.jhin.2005.02.018 16130212

[B4] Scholz HC Hubalek Z Sedlacek I *Brucella microti *sp. nov., isolated from the common vole *Microtus arvalis* Int J Syst Evol Microbiol 2008 58 375 382 10.1099/ijs.0.65356-0 18218934

[B5] Ross HM Foster G Reid RJ Jahans KL Macmillan AP Ross H *Brucella *species infection in sea-mammals Vet Rec 1994 134 359 8017020 10.1136/vr.134.14.359-b

[B6] Ewalt DR Payeur JB Martin BM Cummins DR Miller WG Characteristics of a *Brucella *species from a bottlenose dolphin (*Tursiops truncatus*) J Vet Diagn Invest 1994 6 448 452 7858024 10.1177/104063879400600408

[B7] Cloeckaert A Verger JM Grayon M Classification of *Brucella *spp. isolated from marine mammals by DNA polymorphism at the omp2 locus Microbes Infect 2001 3 729 738 10.1016/S1286-4579(01)01427-7 11489421

[B8] Paulsen IT Seshadri R Nelson KE The *Brucella suis *genome reveals fundamental similarities between animal and plant pathogens and symbionts Proc Natl Acad Sci USA 2002 99 13148 13153 10.1073/pnas.192319099 12271122 PMC130601

[B9] DelVecchio VG Kapatral V Redkar RJ The genome sequence of the facultative intracellular pathogen *Brucella melitensis* Proc Natl Acad Sci USA 2002 99 443 448 10.1073/pnas.221575398 11756688 PMC117579

[B10] Halling SM Peterson-Burch BD Bricker BJ Completion of the genome sequence of *Brucella abortus *and comparison to the highly similar genomes of *Brucella melitensis *and *Brucella suis* J Bacteriol 2005 187 2715 2726 10.1128/JB.187.8.2715-2726.2005 15805518 PMC1070361

[B11] Chain PS Comerci DJ Tolmasky ME Whole-genome analyses of speciation events in pathogenic Brucellae Infect Immun 2005 73 8353 8361 10.1128/IAI.73.12.8353-8361.2005 16299333 PMC1307078

[B12] Wattam AR Williams KP Snyder EE Analysis of ten *Brucella *genomes reveals evidence for horizontal gene transfer despite a preferred intracellular lifestyle J Bacteriol 2009 191 3569 3579 10.1128/JB.01767-08 19346311 PMC2681906

[B13] Audic S Lescot M Claverie JM Scholz HC *Brucella microti*: the genome sequence of an emerging pathogen BMC Genomics 2009 10 352 10.1186/1471-2164-10-352 19653890 PMC2743711

[B14] Crasta OR Folkerts O Fei Z Mane SP Evans C Martino-Catt S Bricker B Yu G Du L Sobral BW Genome sequence of *Brucella abortus *vaccine strain S19 compared to virulent strains yields candidate virulence genes PLoS One 2008 3 e2193 10.1371/journal.pone.0002193 PMC2364660 18478107

[B15] Rajashekara G Glasner JD Glover DA Splitter GA Comparative whole-genome hybridization reveals genomic islands in *Brucella *species J Bacteriol 2004 186 5040 5051 10.1128/JB.186.15.5040-5051.2004 15262941 PMC451633

[B16] Lamontagne J Butler H Chaves-Olarte E Extensive cell envelope modulation is associated with virulence in Brucella abortus J Proteome Res 2007 6 1519 1529 10.1021/pr060636a 17343405

[B17] Lamontagne J Forest A Marazzo E Intracellular adaptation of Brucella abortus J Proteome Res 2009 8 1594 1609 10.1021/pr800978p 19216536 PMC2771391

[B18] Yu CS Lin CJ Hwang JK Predicting subcellular localization of proteins for Gram-negative bacteria by support vector machines based on n-peptide compositions Protein Sci 2004 13 1402 1406 10.1110/ps.03479604 15096640 PMC2286765

[B19] Bhasin M Garg A Raghava GPS PSLpred: prediction of subcellular localization of bacterial proteins Bioinformatics 2005 21 2522 2524 10.1093/bioinformatics/bti309 15699023

[B20] Gardy JL Laird MR Chen F Rey S Walsh CJ Ester M Brinkman FSL PSORTb v.2.0: expanded prediction of bacterial protein subcellular localization and insights gained from comparative proteome analysis Bioinformatics 2005 21 617 623 10.1093/bioinformatics/bti057 15501914

[B21] Connolly JP Comerci D Alefantis TG Proteomic analysis of Brucella abortus cell envelope and identification of immunogenic candidate proteins for vaccine development Proteomics 2006 6 3767 3780 10.1002/pmic.200500730 16739129

[B22] Klinke S Zylberman V Bonomi HR Haase I Guimarães BG Braden BC Bacher A Fischer M Goldbaum FA Structural and kinetic properties of lumazine synthase isoenzymes in the order Rhizobiales J Mol Biol 2007 26 664 680 10.1016/j.jmb.2007.08.021 17854827

[B23] Zylberman V Klinke S Haase I Bacher A Fischer M Goldbaum FA Evolution of vitamin B2 biosynthesis: 6,7-dimethyl-8-ribityllumazine synthases of *Brucella* J Bacteriol 2006 188 6135 6142 10.1128/JB.00207-06 16923880 PMC1595393

[B24] Robertson GT Roop RM Jr The *Brucella abortus *host factor I (HF-I) protein contributes to stress resistance during stationary phase and is a major determinant of virulence in mice Mol Microbiol 1999 34 690 700 10.1046/j.1365-2958.1999.01629.x 10564509

[B25] Bellefontaine AF Pierreux CE Mertens P Vandenhaute J Letesson JJ De Bolle X Plasticity of a transcriptional regulation network among alpha-proteobacteria is supported by the identification of CtrA targets in *Brucella abortus* Mol Microbiol 2002 43 945 960 10.1046/j.1365-2958.2002.02777.x 11929544

[B26] Manterola L Guzmán-Verri C Chaves-Olarte E Barquero-Calvo E de Miguel MJ Moriyón I Grilló MJ López-Goñi I Moreno E BvrR/BvrS-controlled outer membrane proteins Omp3a and Omp3b are not essential for *Brucella abortus *virulence Infect Immun 2007 75 4867 4874 10.1128/IAI.00439-07 17664262 PMC2044513

[B27] Tibor A Wansard V Bielartz V Delrue RM Danese I Michel P Walravens K Godfroid J Letesson JJ Effect of omp10 or omp19 deletion on *Brucella abortus *outer membrane properties and virulence in mice Infect Immun 2002 70 5540 5546 10.1128/IAI.70.10.5540-5546.2002 12228280 PMC128365

[B28] Essenberg RC Sharma YK Cloning of genes for proline and leucine biosynthesis from *Brucella abortus *by functional complementation in *Escherichia coli* J Gen Microbiol 1993 139 87 93 8450311 10.1099/00221287-139-1-87

[B29] Castañeda-Roldán EI Ouahrani-Bettache S Saldaña Z Avelino F Rendón MA Dornand J Girón JA Characterization of SP41, a surface protein of *Brucella *associated with adherence and invasion of host epithelial cells Cell Microbiol 2006 8 1877 1887 10.1111/j.1462-5822.2006.00754.x 16817909

[B30] Valderas MW Alcantara RB Baumgartner JE Bellaire BH Robertson GT Ng WL Richardson JM Winkler ME Roop RM Role of HdeA in acid resistance and virulence in *Brucella abortus *2308 Vet Microbiol 2005 107 307 312 10.1016/j.vetmic.2005.01.018 15863292

[B31] Essenberg RC Cloning and characterization of the glucokinase gene of *Brucella abortus *19 and identification of three other genes J Bacteriol 1995 177 6297 6300 7592399 10.1128/jb.177.21.6297-6300.1995 PMC177474

[B32] Wang R Prince JT Marcotte EM Mass spectrometry of the M. smegmatis proteome: protein expression levels correlate with function, operons, and codon bias Genome Res 2005 15 1118 1126 10.1101/gr.3994105 16077011 PMC1182224

[B33] Brunner E Ahrens CH Mohanty S Baetschmann H Loevenich S Potthast F Deutsch EW Panse C de Lichtenberg U Rinner O Lee H Pedrioli PG Malmstrom J Koehler K Schrimpf S Krijgsveld J Kregenow F Heck AJ Hafen E Schlapbach R Aebersold R A high-quality catalog of the *Drosophila melanogaster *proteome Nat Biotechnol 2007 25 576 583 10.1038/nbt1300 17450130

[B34] Gupta N Tanner S Jaitly N Adkins JN Lipton M Edwards R Romine M Osterman A Bafna V Smith RD Pevzner PA Whole proteome analysis of post-translational modifications: applications of mass-spectrometry for proteogenomic annotation Genome Res 2007 17 1362 1377 10.1101/gr.6427907 17690205 PMC1950905

[B35] Merrihew GE Davis C Ewing B Williams G Käll L Frewen BE Noble WS Green P Thomas JH MacCoss MJ Use of shotgun proteomics for the identification, confirmation, and correction of *C. elegans *gene annotations Genome Res 2008 18 1660 1669 10.1101/gr.077644.108 18653799 PMC2556273

[B36] Mandel MJ Stabb EV Ruby EG Comparative genomics-based investigation of resequencing targets in *Vibrio fischeri*: focus on point miscalls and artefactual expansions BMC Genomics 2008 9 138 10.1186/1471-2164-9-138 18366731 PMC2330054

[B37] Deshayes C Perrodou E Gallien S Euphrasie D Schaeffer C Van-Dorsselaer A Poch O Lecompte O Reyrat JM Interrupted coding sequences in *Mycobacterium smegmatis*: authentic mutations or sequencing errors Genome Biol 2007 8 R20 10.1186/gb-2007-8-2-r20 17295914 PMC1852416

[B38] Aragón V Díaz R Moreno E Moriyón I Characterization of *Brucella abortus *and *Brucella melitensis *native haptens as outer membrane O-type polysaccharides independent from the smooth lipopolysaccharide J Bacteriol 1996 178 1070 8576040 10.1128/jb.178.4.1070-1079.1996 PMC177767

[B39] Moriyon I Berman DT Effects of nonionic, ionic, and dipolar ionic detergents and EDTA on the *Brucella *cell envelope J Bacteriol 1982 152 822 6813315 10.1128/jb.152.2.822-828.1982 PMC221536

[B40] Lucero NE Jacob NO Ayala SM Escobar GI Tuccillo P Jacques I Unusual clinical presentation of brucellosis caused by *Brucella canis* J Med Microbiol 2005 54 505 508 10.1099/jmm.0.45928-0 15824432

[B41] Dricot A Rual JF Lamesch P Bertin N Dupuy D Hao T Lambert C Hallez R Delroisse JM Vandenhaute J Lopez-Goñi I Moriyon I Garcia-Lobo JM Sangari FJ Macmillan AP Cutler SJ Whatmore AM Bozak S Sequerra R Doucette-Stamm L Vidal M Hill DE Letesson JJ De Bolle X Generation of the *Brucella melitensis *ORFeome version 1.1 Genome Res 2004 14 2201 10.1101/gr.2456204 15489343 PMC528937

