# Supplementary material for: Proteomics-based confirmation of protein expression and correction of annotation errors in the Brucella abortus genome
Source: BMC Genomics. 2010 May 12;11:300. doi: 10.1186/1471-2164-11-300 (PMC2877026; doi:10.1186/1471-2164-11-300)
Supplement: Additional file 1 — Proteins newly demonstrated in B. abortus 2308. Each entry is represented by a gene locus tag, description of the protein and the sequences of the peptides measured. Proteins are organized by predicted subcellular localization. [file 1471-2164-11-300-S1.PDF]

Additional File 1. Proteins newly demonstrated in *B. abortus* 2308. Each entry is represented by a gene locus tag, description of the protein and the sequences of the peptides measured. Proteins are organized by predicted subcellular localization.

Locus tag	Protein description	Peptide sequence
**Cytoplasm**		
BAB1_0002	DnaN	AELEAPSGTEGMPGIIIPR + m12|1 Oxidation (M)
		FTVGSVVLTSK
		LIDGTFPDYQR
BAB1_0022	Unknown	DPIISPYTGISYPR
		FYDLNR
		FYDLNRDPIISPYTGISYPR
BAB1_0023	AroA	LAGGEDVADLR
BAB1_0035	KdsB	TVPLGVDTQADLDR
BAB1_0063	Unknown	AAGLPLPEDLVELEK
BAB1_0071	ArgG	FELSAYALNPDIK
		HVEGEVTLK
		KGEAPFSVDANLLHSSSEGK
		TQLLEFAEQHQIPVAK
BAB1_0100	Putative AsnC family	IYIAENEDIGK
		FINENVLDIPNIVR
BAB1_0107	Trs-ABC (P-loop)	VLLLDEPFGALDAQVRK
BAB1_0118	Unknown	AADRDLHSGFFGGAAANPIHILTK
		GLVGEEIVIK
		TAESFLGPIGLSIPAGEK
BAB1_0122	GyrB	QITGYADSSGMTK
		SLLEEPIYIR
		LMGDEVEPR
BAB1_0139	NifU	HFVPEVQQVEQI
		HGIQNLLR
BAB1_0159	S30EA	NAGNDEVNIVYR
		YFDHGFSGQVTVAK
BAB1_0160	PtsN-like	AAELTGLPEREVFETVLQR
		MDLSDLIQPGAIIPALK
BAB1_0191	GABAtrnsam	IGTLGTGFTASGHPVAAAVALENLAIIEER
		LRELQDHPLVGEVR
		SHGPVIDLAEK
		YHLHSYTDAVR
		TGLEQPGALGAR
		LEAEGPLVIER
BAB1_0204	AdhP	AFEQALGMVAR + m8|1 Oxidation (M)
BAB1_0215	ThiE	IGNIPLVGIGGLSVER
		LGVSSHDEAELDR
BAB1_0216	ThiG	LAYEADPIEAR
		LLLGTAQYPSPSILADAVR
BAB1_0242	ManR	EALVEDALQAK
		GACSIVQVDVAR
		IGGITPWLK
		LLADHLAPLVIGEDADCIEAIWR
BAB1_0285	HisD	DFEDAIPLANR
BAB1_0317	Trs arginine/ornithine	APEGGMVLFHVAPGDVVEAGAK
		LATVVTRPGEPEGDIAITAPQAGR
BAB1_0331	ArgD	HIETAVER
		LLPPLITTPEEAR
		TLATIAAGGQAK
BAB1_0344	Pip	GFLESDDQLLR
BAB1_0353	Unknown	GVNDALIAFQR
BAB1_0364	Saccharopine dehydrogenase	WFSTNFDPEINFR
		LAADDYLDEVK
BAB1_0416	DUF85	RFEIAAR
BAB1_0429	Polyprenyl synthetase	ITLPVILSYR
BAB1_0446	DnaJ	FVEDGQTIR
		IMLPEGGDK + m2|1 Oxidation (M)
BAB1_0447	FabI-1	TLAASGIGDFR
		AVDAPDISVVKD
BAB1_0482	FabD	ALAEQFAEAR
BAB1_0484	AcpP	KIVVEHLGVDADK
		IVVEHLGVDADK
BAB1_0489	Guanylate kinase	AISSVENGVAR + m7|1 Deamidation (N)
BAB1_0510	ThrC	GEAPVLGFSDALLAGLAR

Locus tag	Protein description	Peptide sequence
**Cytoplasm**		
BAB1_0525	PpdK	DDAAGFLTTYQNR
		VMPEAFAEFLK + m2|1 Oxidation (M)
BAB1_0532	Transthyretin	GAELAHEPFLDLIPIR
		TDAPLLSGDEMR
BAB1_0540	Formyl transferase, N-terminal	KGSVNLHPSLLPAYR
BAB1_0544	DegT/DnrJ/EryC1/StrS aminotransferase	TITTGEGGMVLAR
		TITTGEGGMVLAR + m9|1 Oxidation (M)
BAB1_0561	Mannose-6-phosphate isomerase type II	GVAEIVIDDK
BAB1_0570	XylA	EGYETLLNTDLSR
		HYNADEIVLGK
		STGFFGDIQK
		YAGWNGEFGK + m5|1 Deamidation (N)
		YLNEIVDIFEK
BAB1_0587	Unknown	EKAGDEPVDAVVGAIPGLGQFV
		ITSNVGIDAATAEK
		MEELIAR
		AGDEPVDAVVGAIPGLGQFV
		VQQLLAAIPGAEEAVSQVK
BAB1_0588	ATP/GTP-binding (P-loop)	TAEDAIFLGASR
BAB1_0641	Membrane alanine aminopeptidase:Neutral zinc metallopeptidases, zinc-binding region	ATALAVLVHR
		IAGAGKLSTDLR
BAB1_0666	DapA	GSITALVTPFDREGAFDEK
		SPMVTIEAATAEK
		ALFLEPNPSGPK
		SISIPLVIYNIPGR
BAB1_0671	RpoZ	FELVLLAGHR
		MSEEELLAGIEGLVAPEK
		QISQGAPITVDR
		EIADETLSPDDLK
BAB1_0688	PyrC-1	ALGNLIQMNPPVR + m8|1 Oxidation (M)
		DVAELEHLPGAAGIK
		IAVGYDADLTIVDMK
BAB1_0697	CysS	DYPDLPFNEAIR
BAB1_0718	MoaD	IGKGEEFIELPAEAVSVGELIAR
BAB1_0740	Unknown	ELSIEPITLDYDGAQK
		SDGGDVGDVVR
		SVIGDVLFMR
BAB1_0775	AspS	ELPLPVFGEPDYPEDIR
BAB1_0780	HemB	LGIPAIAPFPR
		TDDGSFVANPDNLINR
BAB1_0787	GlyA	AVLEAQGSILTNK
		GGMILTNDADIAK
		GGMILTNDADIAK + m3|1 Oxidation (M)
		LILAGGTAYSR
		WFNVVSYGVR
		KDDHLLDMDEVAR
BAB1_0789	RibD	VVVAATDPDER
BAB1_0790	RibE	HSLEVTTWGER
		LTTISSWQSGR
		MFTGIITDIGKVDR
		MFTGIITDIGKVDR + m1|1 Oxidation (M)
		APEELAPFIAQK
		MFTGIITDIGK
		VKPLNEGVLLR
		VNIEIDQLAR
BAB1_0813	CysD	FIGGHGNSMGGILVDGGTFDWAK + m9|1 Oxidation (M)
		QLYGGSINQFGQSFK
		SPGIETLAVHAGAKPDPATGAR
		VAAGAGPDVVR
		ITNPTTAVLEER
BAB1_0817	Unknown; conserved	GSESGITLGSR
BAB1_0826	NuoE	DAYEDLTPER
		LADDAVQPATFAFNAENEAWAHK
BAB1_0842	ProS	FATMDLIGLPTQVIVGPR
		GVAAGEVEVK
		LYEALTNAGVDPLLDDKDERPGAK
		YFLPILK

Locus tag	Protein description	Peptide sequence
**Cytoplasm**		
BAB1_0855	GRX family	EYSSWPTIPQLYVK
		GVNVLASDELR
		NNDVVLFMK + m8|1 Oxidation (M)
		TGINDIIDNEVK
		NNDVVLFMK
BAB1_0856	BolA-related	AMDAHEIEK + m2|1 Oxidation (M)
		GNMGGVLHALALQTSVPE + m3|1 Oxidation (M)
		VQQHQMVYDALK
		VTIRDLAGDGDHFAAEVVAESFR
		GNMGGVLHALALQTSVPE
		DLAGDGDHFAAEVVAESFR
BAB1_0857	FGAM synthase II	GATLDAGDGLPHALLFGEDQAR
		RYNGNILVNAFAAGLAR + m3|1 Deamidation (N)
BAB1_0861	PurS	AIVGALGSLGFDGVSSVR
		VFDLELEGSDK
BAB1_0864	HpcH/HpaI	DAYDALDAILDVR
		FLTISNDSGYIR
BAB1_0874	AcpP	IPLEQWTQEVNEGK
		IPLEQWTQEVNEGKVPTEEYFVLK
		VADIIAETSEIDR
		IDELVAAK
		VPTEEYFVLK
BAB1_0880	HAD-like	WAIVTSAPLELAR
BAB1_0886	NN:DBI PRT	NLPGPDLGAER
BAB1_0896	ArgS	VNDPDLSLAR
BAB1_0898	NagZ	AAAEGLLAGGVLPVVK
		VSVALNELVAHDFVPFK
BAB1_0918	GatB/Yqey	FVGQAIEYEAR
		GQFEDVEIGIER
		SGVALMEIVSKPDLR + m6|1 Oxidation (M)
BAB1_0924	AccC	ALDEFVVDGVK
		LQVEHPVTEAITGIDLVHEQIR
		RLGIPVVPGSDGGVTDEVEAAR
		SAEELPIALATAR
		YLEKPR
BAB1_0933	PCRF 2	SYVLQPYQLVK
BAB1_0943	TyrS	INDEPVSDPR
		LYTTLPLDEIAR
BAB1_0949	SufC	STLSYILAGR
BAB1_0955	DeaD	AFTIVTSSDTK
		DVLGIAQTGTGK
		TASFVLPMLTLLEK
		TTFAELGLSPK
BAB1_0960	Trs heavy metal	LDAEAPVVVDLANK
BAB1_1014	MetG	SVGNVIDPFELVER
BAB1_1030	Gor	SGAIEVDDYSR
BAB1_1037	Mandelate racemase; muconate lactonizing	ATVVDLDGPLLLAR
BAB1_1043	Unknown	AAEPVGNEPVVK
		ITMELGSVEAR
		LTDELGTIEAR
BAB1_1050	FolB	YLIETLALDVAK
BAB1_1075	SseA-2	AGYPVTDEVTK
		AHIPGAVFFDQDK
		DAVVDFQEMR
		NVPVTTLSENGELK + m10|1 Deamidation (N)
		IAATFFKPSFNK
BAB1_1077	Ach1p	LLTEAHILSR
		IGLPYIPIAPEK
BAB1_1096	NifU-like	ILEFAGNMER
BAB1_1098	PRA-CH	FDASGLITAIVTDAR
BAB1_1121	DNA gyrase subunit A	EAIIITEIPYQVNK
		LFVANTHTPVLFFSSR
		NGINSAYTTGR
		RVPLTTYR
		YHPHGDASIYDALVR
BAB1_1130	ClpA/B	ALEEVMGVDK
		DLAEIPDNVK
		IVEYLAVQAR
		SVVPDFENYVK

Locus tag	Protein description	Peptide sequence
**Cytoplasm**		
BAB1_1132	ClpP	LNEIYVK
		RLNEIYVK
		TLDRDHFMTAQEALEFGLIDK
		HAQDIIK
		IMVHQPSGGFQGQASDIER
BAB1_1156	KdsA	GVSFGYNTLVSDMR
BAB1_1157	PyrG	SIGIAPDILLVR
BAB1_1161	TpiA	AAETLEGETVGLGGQDAHFK
BAB1_1164	TrpC	AAGQFALIAEIK
		DFLFDPYQVYEAR
		LAKMAPSDR + m0|1 WMA +1 (N-term)
BAB1_1169	GltX	DLDDFIILR
BAB1_1170	GltA	DAHPMAVMVGCLGAMSAFYHDSTDITDPHQR
		DDPLLDVAMELER
		IFILHADHEQNASTSTVR
		LMGFGHR
		TASFTLDGK
		TFDLPVRK
		GTVGPDVVDIGPLYK
		IALTDEYFIEK
BAB1_1174	FabZ	IVDIDGDVSATGIK
		LEAADIQALLAVLPHR
		RPVVPGDR
		YPFLLIDR
BAB1_1187	Endoribonuclease L-PSP	ACVINVLAQAK
		LVHTGLIGK
		ELTVADGQAAAR
BAB1_1188	GDPD	VPLVIELK
BAB1_1205	ElaB-domain	AEDAMDEASGR + m5|1 Oxidation (M)
		ARAEDAMDEASGR
		ARAEDAMDEASGR + m7|1 Oxidation (M)
		GQVVAEAVR
		AEDAMDEASGR
		SLASHSDDLK
		AEANINDIQQALEK
BAB1_1212	BhbA	ILFDGIPLEK
BAB1_1213	Unknown; conserved	LYMVEFVTEGEER
BAB1_1223	AlaS	IAAVLQGVHDNYDIDLFK
		AADAIAAVK
BAB1_1224	RecA	ALDAALSQIER
BAB1_1233	RpsM; S13	EAIDADYQVEGDLR
		VNIALQYIHGIGPK
		VNQLSDAEVLQIR
		IAGVNIPTNK
		RVNIALQYIHGIGPK
BAB1_1234	Adk	AAVAQQSEIGK
		AVMDAGQLVSDEIVNQIVSER
		AVMDAGQLVSDEIVNQIVSER + m3|1 Oxidation (M)
		IINGMAPVEEVTAEIER + m3|1 Deamidation (N)
		IINGMAPVEEVTAEIER + m3|1 Deamidation (N); m5|1 Oxidation (M)
		LDAVIELK
		LILLGPPGAGK
		TAPLSSYYAGTGELR
		VDENALVK
		RLVEYR
BAB1_1241	RpsH; S8	AEIEIELK
		GVMADHEAR
		RVYVSVK
		SIPQVANGLGISILSTPK
		SVSDPLGDMLTR
		SVSDPLGDMLTR + m9|1 Oxidation (M)
		YYEGVPVIR
		VLDVLQAEGYIR
BAB1_1242	RpsN; S14	AIVMDQGLPLEER + m4|1 Oxidation (M)
		AIVMDQGLPLEERFR
		AIVMDQGLPLEER
		QLGSLGQIPGVVK

Locus tag	Protein description	Peptide sequence
**Cytoplasm**		
BAB1_1244	RplX; L24	KGDSVVVLSGK
		VMPKDEQALVSGINIVK
		DEQALVSGINIVK
		QTQTQEAGIISK
		EAPIHLSNLAIADPK
BAB1_1245	RplN; L14	FDNNAAVLIDNK
		FDNNAAVLIDNKK
		RPDGSVIR
BAB1_1248	RplP; L16	VMFELDGVPEDVAR + m2|1 Oxidation (M)
		VMFELDGVPEDVAR
		GGTDLNFGAFGLK
BAB1_1249	RpsC; S3	ADIDYGTAEAK
		KLSEMTNADTSLNIVEVR
		KPEVDATLIAQSIAQQLER
		LLHEDVK
		VPLHTLR
		IREFLTEELK
		WYANTGEYGK
BAB1_1256	RpsJ; S10	EQFEMR
		GPIPLPTR
		LLDIVDPTPQTVDALMK
		LLDIVDPTPQTVDALMK + m16|1 Oxidation (M)
		SREQFEMR
		RLLDIVDPTPQTVDALMK
		RLLDIVDPTPQTVDALMK + m17|1 Oxidation (M)
BAB1_1266	RplJ; L10	ESGSVVVAHYTGLTVAQMSDLR
		LVGMIQTPAQR
		LVGMIQTPAQR + m4|1 Oxidation (M)
		SLASLPSLDELR
		LAVLTSAPAGQIAR
BAB1_1280	Unknown	KLDAYFK
		TFNNPDLQVK
BAB1_1286	GloA	APELELTYNWDPETYTGGR
		LGLEEIR
BAB1_1294	Aminotransferase	DVLVESFGR
BAB1_1297	Unknown	FAVEFTPEHGTIDLGEGR
		LTATVEASQENLASLEDVVASHIVR
		YLQQLCK
		VVILDADAEK
BAB1_1376	UreA	TVEEVMDGAR
BAB1_1408	IlvB	DVIMPQYAIER
BAB1_1449	UDP-N-acetylmuramate--L-alanine ligase	AENLGDAEVIVVSTAIKK
		MDGAASLFDVVIR
BAB1_1508	CarB	FLEANGVESTK + m5|1 Deamidation (N)
		LGLSVEQVHDASK
BAB1_1512	CspA	VSYEIVQDR
		VSYEIVQDRR
		AGLTTLDEGQK
BAB1_1523	GreA	AEVIDVSK
		AHGDLSENAEYHAAK
		GEGDTIEVNAPGGSR
		IIEAIAEAR
		ISISSPIAR
		SYEIIALK
		INELEDLVAR
BAB1_1528	SseA-1	DLVTHAEFIMVALDADGKPTPAPQED
		NTENEPTGMLTIR
BAB1_1538	OmpR	SLLSQYLTNSGFR
BAB1_1547	PepQ	NILTELDLLGSR
BAB1_1549	PrsA	GANSVTAYITHGVLSGGAVAR
		ITAVLPYFGYAR
		VLSISDLIGEAIAR
BAB1_1553	YchF	APAAAGVIHTDFER
		DISPEDLLTLK
		EALAILPVMEK + m9|1 Oxidation (M)
BAB1_1613	Unknown	LEGSDTDPR

Locus tag	Protein description	Peptide sequence
**Cytoplasm**		
BAB1_1645	DhaK-1	AGDIVLTMAER
		AYLSEIDGK
		SFAEALEALVAAAEK
		TLLDTLVPAVEAFDEANAAGK
BAB1_1646	DhaK-2	AATGATLEEVR
		NMLDAVAVGELFSSPTAK
		NMLDAVAVGELFSSPTAK + m2|1 Oxidation (M)
		FINNPDEVVEDTVR
		VGVVTGGGSGHEPAFIGYTGK
BAB1_1655	GabD	AHIEDAVSK
BAB1_1669	PAS domain	AHVLPGADGADK
		FGSAVLQR
BAB1_1671	TcaR	LLTGERPEPTFLVTKPFNPDMVK + m21|1 Oxidation (M)
		MPETSSEFAWETHAAR
		LLTGERPEPTFLVTKPFNPDMVK
		TKDEALALYEK
		LLSTASGEISR
		NLAHIAPLPR
BAB1_1687	Dut	IAQAVFAPVIQPK
		VLLINLGDDDFR
		VLLINLGDDDFRIER
		TAASSSAPTLGIIR
		TLVPTGLILEIPQGYEVQIRPR
BAB1_1695	PurA	GQGIDVTPDVLR
		LDVLDGLEEIK
		VAENAPLILSIHR
		LSLLPSGLVR
BAB1_1702	Phosphoglucosamine mutase	LVIRPSGTEPLIR
BAB1_1719	ThiE	IVLVAPPIADGAALAK
BAB1_1722	Efp	IPVPPFIASGER
		VIVDTNELTYISR
BAB1_1742	PgK	GLGNPARPVVAIVGGAK
		TLDDANVQSK
		IVPTIAELSR
		AHASTEGLAHVLPAFAGR
BAB1_1751	Unknown	IALLQSEIER
BAB1_1761	PyK	TPIIALSPVVDTAR
BAB1_1778	FdxA	TYVVTDNCIR
		WPNITAK
		WLELNAEYAAK
BAB1_1781	Unknown	AGYGDVVNVLAAGR
BAB1_1804	MarR family	TLLALIEQEGLYQK
BAB1_1810	AtpH	AETSSLISGVAQR
		YAGSLFEHALDANSVASVEK
BAB1_1813	Transaldolase	EAALIGADVVTAPPATLK
		GPVSAEVAATEYEQMMK
		LDDTGINGMELIAEIR
		LDDTGINGMELIAEIR + m7|1 Deamidation (N)
		TIYDNYDFR
		VNMTLCFSANQALLAAK
		AGATFISPFIGR
		ELNDLGLVDGVTTNPSLILK
		TEILAASVR
		GLETFLADWAK
		FFVDTADVK
BAB1_1815	LeuS	RGDLTIAR
		SGALVEQR
BAB1_1819	Acetyl-CoA synthetase	ALMGAGDEFVTR
		IQFAPGLPK
BAB1_1824	PurH	NHAYVATVVDPADYADVVAELEK
		TLDEEAAEEIVK
		TLHPAVHGGLLAVR
		YGENPHQTAGFYLTGEK
		LLVTGGLPDPR
BAB1_1837	CynT	AAALDTESAPLSPSDFIGK
		YRDLAEK
		YSLANLR
		AAPETIFNAAPGEIFVLR

Locus tag	Protein description	Peptide sequence
**Cytoplasm**		
BAB1_1840	MmsA	ASGFGDLNQHGPDAFR
		DGAEFVIPTMK
		DGAEFVIPTMK + m10|1 Oxidation (M)
BAB1_1872	PrfA	ALAVEELPEVEK
BAB1_1874	LysC	ISVIGIGMR
		TDMTFTIPTGDIDK + m3|1 Oxidation (M)
BAB1_1879	GrxC	KGAEFNEIDASATPELR
BAB1_1887	HemC	GLFTEEIEQALK
BAB1_1895	FtsK-gamma	VIGLADDIAR
BAB1_1918	LpdA-2	ALLHASEVFAEAGHSFDTLGVEVTPK
		ANVSGVEFLFK
		ANVSGVEFLFKK
		ESALATFAKPIHM
		ESALATFAKPIHM + m13|1 Oxidation (M)
		ITPYIGTGK
		NIIIATGSDVAGIPGVK
		SYDVVVIGTGPGGYVAAIK
		TFGGTCLNIGCIPSK
		TNVEGIYAIGDVVQGPMLAHK
		TNVEGIYAIGDVVQGPMLAHK + m17|1 Oxidation (M)
		VLGPMDGEVSK
		VLGPMDGEVSK + m5|1 Oxidation (M)
		VSVTSEDGKVEEIEAK
		VSVTSEDGKVEEIEAK + m0|1 WMA +1 (N-term)
		VTFEPVK
		VTFEPVKGGDAETLEADAVLIATGR
		VTVVEYLDK
		GGDAETLEADAVLIATGR
		RPYTDGLGLQEAGVAVDER
BAB1_1926	SucC	MNIHEYQAK
BAB1_1936	GloB	GGDEFTFGLFK
		LGLQDAPDEAVFAEIRK
BAB1_1946	SecA	EHVTIQPENQTLASITFQNYFR
		TPLIISGPLEDR
BAB1_1970	FadB	GFYDYRGEHPVPTR
		GIATINGNMAR + m6|1 Deamidation (N)
		SNDFVTALGK
		TVTVAEDFPAFIVNR
BAB1_1971	EtfA	AILLIAEHDNATLSDQTAK
		FQEVILPVADK
		VLLAESDALENR
BAB1_1988	HisC	AGVLDIAAYVPGK
		GVVAALTEFLK
		IDVDAILAGVTAR
BAB1_1993	Ppa	AGALIVDR
		IHDYTDMPEITLK + m7|1 Oxidation (M)
		KFIVEAIER
		IGDWGDEDYAR
		KAGALIVDR
		FIVEAIER
BAB1_2006	RegA	DPSNATLLVTESGGYK
BAB1_2016	RpmB; L28	GGLDAFLVK
BAB1_2023	ClpA/clpB	DIANLPDTTVSVR
BAB1_2059	ParB	GLAALIGEIDRPVEER
		MFSETELEDLAQSIK
		SLITTENPTALAER
BAB1_2080	HslU	DSYPILINDESDK
		ILTETEASLIK
		LLDQDQIVQEALR
		VSEDEGIVFIDEIDK
		DLLPLVEGTTVATK
BAB1_2081	HslV	LEQYPDQLMR
BAB1_2087	HisE	LGEEAVETVIAAVSGDR
BAB1_2096	PTS system IIA subunit	IEVIAGVNLPMLIK
BAB1_2109	AccD	FFFDNGEYTTLEAPK + m5|1 Deamidation (N)
BAB1_2133	Unknown	FGANFIAR
BAB1_2134	SMP-30	VDTDPETGLPVSEK
BAB1_2135	Glutathione synthetase	TLVVNDPAWVR

Locus tag	Protein description	Peptide sequence
**Cytoplasm**		
BAB1_2149	PepS	EGQDLVITAPVVALPLVR
		YANAPDESFDR
BAB1_2168	RpsO; S15	IANLTEHFK
		EGDTGSPEVQVAVLSER
BAB1_2173	FabB	TIVDSADITR
		YNDTPSTASR
BAB2_0083	Eda2	GQPVIPVLLIDK
		TDLLLPIMK
BAB2_0090	GCN5-related N-acetyltransferase	SDDPLFFAVIDK
BAB2_0109	Gnd	AMVDMILDEAGQK
		DFFGSHGFK
		FLSDLEQGLLAGK
		IAPILLAVAAK
		SQLLDDIAQAFEGNESR
		TLDIANQTR
		NLLMAPAFVPR
BAB2_0160	Unknown	LFLQQGLFNLK
BAB2_0162	L-carnitine dehydratase/bile acid-inducible protein F	FVSNVLR
BAB2_0177	YafB	DLFMETWR
		ESGVTPVLNQIELHPQFQQDELR
		LLEDPTLK
		LWNSDQGYESTLK
		SIGVSNFR
BAB2_0186	Fumarate hydratase	AAAETNIALGK
		AIELLGGEMGSK
		MPLPLVHALGVVK
BAB2_0187	Unknown	KIEPTAWIR
BAB2_0191	HAD-like, subfamily IIA	ILGIGDGVLTDVK
BAB2_0198	Pseudouridine synthase	EGGDRDKPR
BAB2_0216	3-hydroxybutyryl-CoA dehydrogenase	QQLAEHFR
BAB2_0246	P47K	IPVTVLTGYLGSGK
		TGLDAVVALVDAK
BAB2_0293	Gal	HVMLEKPPGATISEVHALER
		TTSPDIALAIVGLGK
		TTSPDIALAIVGLGK + m0|1 WMA +1 (N-term)
BAB2_0295	DgoK	DAIVVMPGTHAK
		EAGFEPTLER
		GDDGLLHAK
		IANGELADYR + m3|1 Deamidation (N)
		LLGLVHR
		TFMTGELFALLK + m3|1 Oxidation (M)
BAB2_0296	KdgA	AQQAVAAWDK
		LLVSPNVEPEVIRR
		YPLVAILR
		AGFSAADVQER
BAB2_0301	Dat	IVYVNGEYVAAR + m5|1 Deamidation (N)
BAB2_0335	NADH:flavin oxidoreductase/NADH oxidase	ASLFDPITIGDLK
		DFQQGPQPFDYAR
		ENAPLNELDQK
		TYLVHPDGAGEFVPTSEPR
		SELPGIVADYAR
BAB2_0337	RocF	ILGLPVQEGTGR
		LAAPIDPHNVCMLGIR
BAB2_0343	Trx-2	LAADPGDYQAR
		LIGDPVELER
		VGGPSDQEAALAEAIATVK
BAB2_0358	Dcp	GLFLGDYFAR
		SIVEPFLSFSQNR
BAB2_0361	TypA	DNDLEVNVLK
		DQGLAPLFDLVLK
		MIGTILEADPFLGR
		MIGTILEADPFLGR + m1|1 Oxidation (M)
		TTLVDELLK
		VLGQDGSVLENGR + m11|1 Deamidation (N)
BAB2_0365	FbaA	KPDGSVLAMHVIEEIHR
		VDALAIAMGTSHGAYK + m8|1 Oxidation (M)
		LSHDQLLTDPDEAVK
		SYANDIMLR

Locus tag	Protein description	Peptide sequence
**Cytoplasm**		
BAB2_0366	RpiB/LacA/LacB	AALSNNAQIITMGAR
		DRFEVSEISR
		SAGNVNAINEVDAK
		VASAVLDGTYDR
		VAVAGDSAGEGLAK
BAB2_0367	TIM 2	AAAVLEEEVRGALSK
BAB2_0370	EryC	TFADIIGDLGGK
BAB2_0448	Unknown	DRWTLDELTVAAIER
		ILDLAHEALPER
BAB2_0457	FolD	LAEDVVSTVK
		LVGDVDFAEAEK
		DLSGLNAVVIGR
BAB2_0459	Pgl	FVGLYNPAATAETAALAAASR
		HDFISATELAQSLSEAIANK
		LFEVLSR
BAB2_0460	Zwf	AGQLSDPTR
		EHVKPEEVDQAEVEIFLK
		EHVKPEEVDQAEVEIFLKR
		ETVQNLMALR + m7|1 Oxidation (M)
		GYLEDLESDTSNTETFVALK
		HMPLDMSFAESFEDRNPDAYER
		LHYVAVDAK
		RDEVEAAWR
		SDEGWDALK
		VSEIVVTFKPIPHSIFGESAGK
		ALNDTLGSVFREDQIFR
		ETVQNLMALR
		KLIPALYQR
		VIANQLVIR
		SLQPISTHNVEELTVR
		WAGVPFYLR
BAB2_0483	ShuT	NGAPAYILSALGIR
		TIEGIETLADAIAAAGLNK
BAB2_0513	GcvT	AGADLFDESGR
BAB2_0518	PutA	DYETAIHAIGR
		GIYDGPGISIK
		ILHEGGIPADALQLLPGDGR
BAB2_0566	AldA	RVSLELGGK
		SPNIVFADSDLEK
BAB2_0568	Unknown	GDFSATEWEEALSTASER
BAB2_0572	IlvE	KLDYGLVTK
		ALGLEPTLSANDIEALAR
BAB2_0620	Unknown	GILSQLEIDEALR
BAB2_0642	Acyl-CoA dehydrogenase	FVLSAEAQDLAR
BAB2_0644	Metal-dependent hydrolase	EHGATVIANADLASWLGSQGVEK
		AVILIDPFLNGNPGAK
		AVILIDPFLNGNPGAK + m10|1 Deamidation (N)
		VLADPAGTVHSFQA
BAB2_0645	GatC	IAVSEDDAER
BAB2_0646	GatA	DIADMYEQTR + m5|1 Oxidation (M)
		GEAGALEGIPLGVK
		SELTALTIAEAR
BAB2_0851	GuaB	IVAGVGVPQLSAIMSAVEAAQK
		LAIAMAQAGGIGVIHR + m5|1 Oxidation (M)
BAB2_0961	DapA	AGIPVIVGTGAVNTASAVAHAAHAQK
		GYVETQFR
		LFNTWYAEWSK
		SPDFDALVR
BAB2_0976	AldB	NIDLLTDLESK
		NPSDGSDLALIAR
BAB2_0988	ArgB	ELSVADAQALIR
BAB2_0990	Unknown	IPEPGGEAWAAK
		MPLILFGK
BAB2_0991	DapD	EAVEQSLILLDRGEVR
		GGPGQSSWWDK
		LNPMEVIK
		SEVVEGCIVR
		TSINELLRD
		TKPDLASLEK

Locus tag	Protein description	Peptide sequence
**Cytoplasm**		
BAB2_0993	DapE	VALADLEGLTQIYER
BAB2_1009	MgsA	LGSVYDIPMALNR
BAB2_1012	DapB	DIALADNSVR
		ETGAIGFATLR
BAB2_1013	Gpm	ALIMALDGLTPEQILK
		DYGDLSGLNK
		LNADSTVASK
		SYDVPPPGGESLK
		HGQSEWNLK
		QELNTGVPIIYR
		FDIAYTSALSR
**Inner membrane**		
BAB1_0400	Unknown	ALVALSDGLALVR
BAB1_0425	NhaA	AVETEIPVVR
		VTLDPFQPEK
BAB1_0542	WbkC	IMNMVEK + m2|1 Oxidation (M)
		SAGANIEDIHALK
BAB1_1283	DUF192	AMIFILGEMR + m2m9|2 Oxidation (M)
		INFALEVADTDEAR
		LPVDKEQLTFITSTGNK
BAB1_1703	FtsH	LIDEAYAEATR
		NVPLAPNVDIK
		TNTAYHEAGHAIVALNVPK
		VAYGDNQEEVFLGHSVSR
		VTFQDVAGVDEAK
BAB1_1712	MotA; TolQ; ExbB	SPIGLQMR
BAB2_0261	RecA	IVGYLNVPGR
		RLGAVVTATDVRPAAK
BAB2_0709	FtsK-alpha	AEAPVPAVDVK
		VIGLADDIAR
BAB2_0728	CydA	IPWVMGLIGTR + m5|1 Oxidation (M)
		SLTTPIEGIDQLVQNAEK
BAB2_0877	Binding-protein-dependent transport systems inner membrane component	TAPSSRTLR
		TLRAIDK + m0|1 WMA +1 (N-term)
**Periplasm**		
BAB1_0010	Trs-ABC oligopeptide	IESMKPGHSIIWAR
		IYNPFAASLR
		LFSQAGWTLK
		LIDLVVYAK
		WAEQYNFPAVQR
		YSSSLLDEPK
BAB1_0155	OstA-like	AGQMTVYYSK
		NQIMVLQGQK + m0|1 WMA +1 (N-term)
		NQIMVLQGQK
BAB1_0404	Unknown	FLAALDLAAAGKTDEALAAFTDLEK
		LVAEDSLAPANAR
BAB1_0444	PdxH	LFAEWLADAAK
BAB1_0739	ETC complex I subunit region	VVEPLMGYTSSGDMK
BAB1_0776	Unknown	ANGADIILEGASLK + m2|1 Deamidation (N)
		GPLDSMVLYDK + m6|1 Oxidation (M)
		QITVPDLVFNGK + m10|1 Deamidation (N)
		TGDASLDQLEIK
BAB1_0881	Trs-ABC amino acid	YYNTPPGVAAPK
BAB1_1117	PpiB-2	AYKDPENTLVLETTK
		DPENTLVLETTK
		EGAYDGVVFHR + m0|1 WMA +1 (N-term)
		GNVVLELYPDLAPGHVAR + m0|1 WMA +1 (N-term)
		VIDGFMAQTGDVK + m6|1 Oxidation (M)
		EGAYDGVVFHR
		RGEPVSDPDK
		VIDGFMAQTGDVK
		GNVVLELYPDLAPGHVAR

Locus tag	Protein description	Peptide sequence
**Periplasm**		
BAB1_1118	PpiB-1	DGDVALEIRPDLAPK
		EGAYNGVAFHR + m5|1 Deamidation (N)
		LKDGDVALEIRPDLAPK
		VGTGGSNYPDLPAEFSKEPFVR
		VIPGFMAQTGDVK
		VIPGFMAQTGDVK + m6|1 Oxidation (M)
		VVSGMDAVDK
		VVSGMDAVDK + m5|1 Oxidation (M)
		VGTGGSNYPDLPAEFSK
BAB1_1362	LacI	IVVVSHGQASDPFWSVVK
		KLGALLHVGQDEFDAGK
		LGALLHVGQDEFDAGK
BAB1_1413	DegP	GALIAGLIENSGVDNK
		AIEAGDVVIR
BAB1_1890	YciI-like protein	FAGPFLGEDGKPNGSLVVVEAADK + m13|1 Deamidation (N)
		IAASDPYALAGLFK
		LDTRPAHLDYLK
BAB1_1919	Unknown	DGAEAEVLPK
BAB1_1981	TlpA	DASMASFNELK + m4|1 Oxidation (M)
		GFLSEIGIK
BAB2_0374	Unknown	AVDAVEVGNALAADK
		VDGMPEGMVIR
BAB2_0427	Trs-ABC spermidine/putrescine	NLDELLVWAK
		TLYQEPAWR
		YIEYYPTGTGALMK
BAB2_0451	Trs-ABC oligopeptide AppA family	LLPAPYFNETK
BAB2_0593	Trs-ABC amino acid	DAAKAVEAVK + m0|1 WMA +1 (N-term)
		GPVSIDPESR
BAB2_0611	Trs-ABC amino acid	YYTTPLAVVVPK
		DSDIVSLEPSAFAGK
BAB2_0664	Trs-ABC peptide	LYDFPAVEQGK
BAB2_0697	Unknown; conserved	HQEIRDYK
BAB2_0812	Trs-ABC oligopeptide AppA family	LASIASVETPDEK
		LENIVGDLAK
		YFADPAAAIAALR
BAB2_0879	Trs-ABC spermidine/putrescine	EIYFKPFAEK
		VPEEYAADLPTAK
BAB2_0880	Unknown	NAIWGEAQAATPTAETPAATPSTPAAPVPAAR
		SAAPASGSVDVGAILDAAVKK
		SGQALNWK
		ALGLDSSLQHR
		DSGGKLPAGL
		SAAPASGSVDVGAILDAAVK
		TSIVDLMK
BAB2_1109	XylF	DRDYFIAAAEK
		IVGSQWVK
		LVDGTQTVTVYKPLK
		TAVSGQDSDLAAVK
		VFDAVVADAK
		DVDTLLLTPTAVTK
**Outer membrane**		
BAB1_0659	Omp2a	GGDDVYSGTDRNGWDK + m12|1 Deamidation (N)
		GGDDVYSGTDR
BAB1_0707	OstA	IYSDHIDVTDSFR
		NPGFSGTNLR + m0|1 WMA +1 (N-term)
		SGFLFPGFAYK
		TFITPSGLVITPLLALR
BAB1_0963	TolC	IEVDVVQDK
		YAVNAAGYNVK

Locus tag	Protein description	Peptide sequence
**Unknown localization**		
BAB1_0030	Unknown	TQSLTAGLPIPPGFK
BAB1_0170	GrpE	AEAAADEAEGEVDETANR
		AMLLALER
		IAVLEADNTELKDQMLR
		NKPENPDLDQR
		RAEAAADEAEGEVDETANR
		VAAEMENLR + m5|1 Oxidation (M)
		DMLSVSDNLR
		IAVLEADNTELK
		SLSEGVEMTER
		VAAEMENLR
		ALDAIPADALEADSNLK
		AYAITNFAR
BAB1_0389	CcoP	SPDDPETR
BAB1_0413	AtpB	ASAEAVLESK
		IAQDLEQAAR
		LKQDADNAIAAYEQELAQAR
BAB1_0418	Unknown	APVYQDGSAHR
		GNTDYTAPASIK
BAB1_0420	Unknown	APVYQDGSAHR
		LDYTAPASIK
BAB1_0453	Unknown	LPGEFDIGAINPGMK
		VLIIYDIVDHTR
BAB1_0479	RpsR, S18	FLGLLPYVVK
BAB1_0627	Unknown	ATPTMQNGR + m7|1 Deamidation (N); m5|1 Oxidation (M)
BAB1_0650	Unknown	FCGTVMTGEYK
BAB1_0810	RpsI; S9	AESINSLEELGTVAK + m5|1 Deamidation (N)
		AGQFDIVATVAGGGLSGQAGAVR
		TEAAAPVHVQK
		YFARPVLQMILQQPIVASNR
		YFARPVLQMILQQPIVASNR + m9|1 Oxidation (M)
		AESINSLEELGTVAK
		ALTDYEPGLR
BAB1_0830	NDH-1 subunit I	NIAMDAPYR
BAB1_0991	Unknown	ADAINGINLVK
		ADAINGINLVK + m5|1 Deamidation (N)
BAB1_1070	WrbA	EGGAEVTLK
		NFLDQTGGLWAK
		RVPELVPEEVAK
BAB1_1113	Unknown; conserved	LTPGQFVQVR
BAB1_1152	PdhA	GVMAELTGR
		GWATEEELK
		MRSEHDPIEQVK
BAB1_1230	RplQ; L17	AMFANMAASLIEHEQIVTTLPK + m2m6|2 Oxidation (M)
		AGDNAPLAVVEFVERDVDAK
		AMFANMAASLIEHEQIVTTLPK
		AGDNAPLAVVEFVER
BAB1_1232	RpsK; S11	ALQAAGFVITSIR
BAB1_1240	PplF; L6	QQVGQVAAEIR
		KLEISGVGYR
		KPVPVPAGVTGSVEGQTVK
		TMISNIFVGVK + m2|1 Oxidation (M)
BAB1_1260	RpsL; S12	GVLDTQGVK
		LTNGFEVIGYIPGEGHNLQEHSVVMIR + m3|1 Deamidation (N)
		PTVNQLIR
BAB1_1270	SecE	TNPITFFQQVR
BAB1_1341	Unknown	AALIGSETISINGGK + m12|1 Deamidation (N)
BAB1_1384	Cibk	ADSNGTFSYAMPR + m4|1 Deamidation (N)
		VPNPTFVTQVLK
BAB1_1514	AspC	AFLADALSR
		SLTDVLVR
		TLTMNGVSK + m5|1 Deamidation (N); m4|1 Oxidation (M)
		VIETDKDFVTELLETEGVAVVHGSAFGLGPNFR
		IGYAAGPIELIK
		LTAADLEK
		YTPVSGIPQLR
BAB1_1543	DUF526	IEALEAR
		LVTDAAGAAQGVR

Locus tag	Protein description	Peptide sequence
**Unknown localization**		
BAB1_1559	FbcF	GKPVFIR
		NANLPTDAEATDLAR
		TTALGDLKDPNAR
		SGPAPENMHIPQYAFTSDTVIR
BAB1_1641	Unknown	SPADGKFDLK
		VLTQEGFTSWGEIELDDGLWEVEDAR
BAB1_1647	FabG domain	GSFLVTQAVGR
		VAIVTGGASGIGAAISK
		VAVLDISADIAK
BAB1_1693	bZIP	DTEQAAGNPLIAVPSK
		GGTETGSVQR
		GNVVWSVIEESREDGQPAQPAIR
		IADNFFIIWLNDAR
		NTDQSIPASHLIEMVFTVPEGFPGGAIDNVQR
		TAQDTNLSLMR
		GNVVWSVIEESR
BAB1_1728	RpmE; L31	VVMTDGTEYMTR + m10|1 Oxidation (M)
		VVMTDGTEYMTR + m3|1 Oxidation (M)
		VVMTDGTEYMTR + m3m10|2 Oxidation (M)
		ANIHPDYHTIK
		VVMTDGTEYMTR
BAB1_1749	Unknown	AIADGEASQGIK
		GLDPVITGQLSR
BAB1_1768	Unknown	TAGYGVGGAALGALAGGAIGGNGR + m22|1 Deamidation (N)
BAB1_1784	DUF336	LKPLAVSVLDAGGHVK
BAB1_1814	Unknown	ATSNFVLR
		LASIAIDPAGDIFGQEVR
BAB1_1858	RplU; L21	QELTTIR
		GHRQELTTIR
		VAANDLIK
BAB1_1984	LysA	LLIPEVLVDGER
BAB1_2123	RpmI; L35	GTMVLADADAK + m3|1 Oxidation (M)
		GTMVLADADAK
BAB1_2176	YaeC / NLPA lipoprotein	ALLVLADNGLIK
		APYTNILVVR
		FVELDAAQLPR
		ALLVLADNGLIK + m8|1 Deamidation (N)
BAB1_2186	RpsT; S20	AVEPELMR
		AVEPELMR + m7|1 Oxidation (M)
		LEDALLSGDK
		KLEDALLSGDK
BAB2_0207	Unknown	KLDSLEFR
		LLDPNNMTLK + m7|1 Oxidation (M)
		DLQNLDIVGIYPDYESAK
		LLDPNNMTLK
BAB2_0243	YedY	EFDVEELIAK
BAB2_0269	RpsU; S21	DNNVDQALR
BAB2_0351	OsmC-like protein	AKAEELVQAAHIVCPYSHATR
		ISIPAESTVTATVGIGPR
		PILYTTQSTATGGR
		LSVVLDTPK
BAB2_0356	Unknown	EVDGVDYTPDIK
BAB2_0677	Unknown	SQEDFIGWTR
BAB2_0726	YbgT	LAADPTAELALEGNK
BAB2_0869	HlyD	TILDLTEGGR
		DALQAALDAAQANLAK
BAB2_1002	NqoB	FSPFLPVIGGGETK
		LQPVYVGDVAEAVAR
